# Temporal Eating Patterns and Colorectal Cancer: A Systematic Review

**DOI:** 10.1007/s13668-025-00700-w

**Published:** 2025-10-21

**Authors:** Zegeye Abebe, Molla Mesele Wassie, Amy C. Reynolds, Yohannes Adama Melaku

**Affiliations:** 1 Flinders Health and Medical Research Institute, College of Medicine and Public Health, Flinders University, Adelaide, South Australia 5042 Australia; 2 Department of Human Nutrition, Institute of Public Health, College of Medicine and Health Sciences, University of Gondar, 196 Gondar, Ethiopia

**Keywords:** Colorectal cancer, Meal frequency, Mealtimes, Eating occasions, Dinner time

## Abstract

**Purpose of the Review:**

In addition to diet quality, which influences the risk of colorectal cancer (CRC), temporal eating patterns, such as meal frequency, duration, regularity, and timing, may also play an important role. Recent studies have suggest that these eating patterns can influence CRC risk; however, dietary guidelines predominantly emphasise modifying the intake of specific food items to reduce risk and promote overall health. Additionally, comprehensive studies examining the relationship between temporal eating patterns and CRC risk are lacking. This review aimed to synthesise the available evidence on how temporal eating patterns may affect CRC risk and mortality. Following the Preferred Reporting Items for Systematic Reviews and Meta-Analyses guidelines, a systematic literature review was conducted using databases such as Medline, Scopus, Web of Science, CINAHL, and ProQuest, ultimately including 20 relevant articles.

**Recent Findings:**

Higher eating frequency, particularly when involving unhealthy foods, along with skipping breakfast and increased snacking may elevate CRC risk. Furthermore, a short interval between the last meal and bedtime has been associated with an increased risk of CRC and related mortality.

**Summary:**

The findings suggest that a short interval between the last meal and bedtime may increase CRC risk, potentially through mechanisms such as circadian rhythm disruption, oxidative stress, and inflammation. In addtion, higher meal frequency, particularly when coupled with an unhealthy diet, appears to further elevate the risk. Future research should employ standardised definitions and detailed assessment of 24-hour eating patterns to better elucidate their relationship with CRC outcomes.

**Supplementary Information:**

The online version contains supplementary material available at 10.1007/s13668-025-00700-w.

## Introduction

Colorectal cancer (CRC) is a major public health issue, ranking third among cancers in men and second in women [1]. It is the third-leading cause of cancer-related deaths for both sexes [2]. The global burden of CRC is predicted to surge by 47% to over 3.2 million new cases and 1.1 million annual deaths by the year 2040 [3]. This increase is expected due to the economic development of transitioning in low-to-medium health development index (HDI) nations, and lifestyle changes in developed nations [3, 4]. Key contributors to this increase include sedentary behaviour, rising obesity rates, and higher consumption of processed foods and alcohol [5–7].

Poor diet quality is one of the contributing factors to CRC burden. It contributed to 32% of CRC deaths and 34% of DALYs in 2019 [8]. Evidence from randomised controlled trials and systematic reviews of observational studies has shown that a diet rich in whole grains, fruits, and vegetables is associated with a decreased risk of CRC [9, 10]. In contrast, a diet rich in red and processed meat, and refined grains is associated with an increased risk [11]. In line with this evidence, dietary guidelines such as those from the World Cancer Research Fund (WCRF) and the American dietary guidelines [12, 13] have recognised diet as a risk factor for CRC.

Focusing solely on the components of an individual’s diet may not provide the complete picture of its relationship with CRC. Chrononutrition, an emerging area in nutritional science, examines the influence of meal timing across the 24-hour day. This includes eating behaviours such as eating duration, meal frequency, timing of the eating window, energy distribution, late-night eating, and meal regularity [14, 15]. Meal time significantly impacts health by influencing metabolism and hormone regulation [16]. Eating in alignment with the body’s biological clock can optimise digestion and improve blood sugar control [17]. This is crucial for preventing metabolic conditions, reducing metabolic stress, and lowering inflammation, all of which are associated with an increased risk of CRC. Studies also suggest that consuming larger meals earlier and lighter meals later supports weight management and regulates appetite hormones like leptin and ghrelin. Together, this can reduce the likelihood of overeating, weight gain, and subsequent CRC risk [18–20]. Conversely, eating late at night can contribute to insulin resistance, slower metabolism, and increased fat storage [21].

The evidence on the relationship between 24-hour temporal eating patterns and the risk of CRC is inconsistent. For example, studies suggest that meal frequency (how many meal occasions an individual has in the 24-hour day) may impact CRC risk. Specifically, having more than three eating occasions in a 24-hour day was associated with an increased risk of CRC [22], while more than six eating occasions were associated with a higher risk of CRC mortality [23]. Yet, a study conducted by Mekary et al. in 2012 found that higher eating frequency (> 5 occasions/day) was associated with a decreased risk of CRC [24]. Findings from prospective studies have indicated that eating frequency does not have a significant association with CRC risk [25, 26]. Consequently, there is a need to better understand the inconsistencies in these findings and clarify the relationship between 24-hour temporal eating patterns and CRC risk.

Although one systematic review has explored the association between eating frequency and CRC risk [27], it focused solely on meal frequency without comprehensively assessing other temporal eating patterns, such as eating duration, energy distribution, or late-night eating. Additionally, the review combined cohort and case-control studies with varying definitions of meal frequency. Inconsistent effect size metrics may have introduced bias, potentially influencing interpretation of the relationship between meal frequency and CRC risk.

To address these gaps, this systematic review synthesised existing evidence on the associations of 24-hour temporal eating patterns, including eating frequency, eating duration, eating windows, energy distribution, meal regularity, and late-night eating with CRC risk and mortality. By synthesising current evidence on how temporal eating patterns influence CRC development, this review seeks to provide insights to guide public health interventions and identify key directions for future research.

## Methods

### Protocol Registration

This systematic review was conducted according to the Preferred Reporting Items for Systematic Reviews and Meta-Analysis (PRISMA) [28] and the protocol was registered in PROSPERO with registration ID CRD42024575331. Initially, we planned to include a meta-analysis; however, due to inconsistencies in the definition and measurement of meal frequency and timing, a meta-analysis was not conducted.

## Study Selection and Inclusion Criteria

Articles needed to be written in English language, and report at least one meal timing indicator variable such as daily eating duration, time between first and last eating episodes, eating window, and day-to-day variability in timing of eating, late night eating, or eating frequency with odds ratio (OR), relative risk (RR) or hazard ratio (HR) and 95% confidence interval (CI). All study designs except case reports, case series, editorial reports, systematic reviews, and meta-analyses were included. A complete summary of eligibility according to the participants, exposure, comparison, outcomes, and study design (PECOS) criteria for inclusion can be found in Table 1.

**Table 1:** Participants, exposure, comparison, outcomes, and study design (PECOS) inclusion criteria for studies

Criteria	Population	Exposure	Comparator	Outcome	Study design
Inclusion	≥ 18 years Human participants	- Temporal 24-hour eating patterns (meal timing, duration, regularity, energy distribution, late-night eating, meal frequency, breakfast skipping) - The highest eating frequency/longer time interval/higher energy distribution were taken as exposure	- Different number of daily meal frequencies/time intervals between dinner and bedtime. - It depends on the classification and selection of the reference category by the primary study author’s definition (the lowest meal frequency/shorter time interval was the comparator)	Studies that report risk or mortality of CRC; or colon, or rectal cancer, or both as outcomes	- Cross-sectional - Case-control - cohort and - Randomised clinical trial
Exclusion	- < 18 years - Animals studies	- Studies that only examine the frequency of intake of a single food, beverage, or category of foods or beverages - Studies that do not include meal timing indicator variables	None	Non-colorectal outcomes	- Case-report - Cases series, - Editorial comments - Systematic review and meta-analysis

## Search Strategy

All published studies up to July 8, 2024, were searched across five databases: Medline, Scopus, Web of Science, CINAHL, and ProQuest. In Medline, a master search term was created using free-text words and Medical Subject headings, such as colorectal cancer, colon cancer, rectum cancer, and meal timing, meal frequency, eating frequency, eating occasion, late eating, meal timing, feeding pattern, meal skipping, evening meal, late night eating and dinner to bedtime. A search strategy was first developed in Medline, followed by a master search strategy for translation into other databases (Supplementary Table 1). The primary author (ZA) reviewed titles, abstracts, and full texts using the predefined inclusion criteria and was cross-checked by two senior co-authors (MMW and YAM). Google and Google Scholar were searched for additional studies and grey literature. Reference lists of included studies were checked to retrieve any missing studies.

## Data Extraction

The following study characteristics were recorded: the first author’s name, year of publication, country, study design, meal timing indicator variable and its definition, dietary intake measurement tool, frequency of data collection, meal regularity, recall period, sample size, age and sex of the study participants, methods of measuring the association between exposure and outcomes, confounders considered during analysis, and the main conclusion of each included study. Data were independently extracted by the primary author (ZA) and cross-checked by the co-authors (MMW and YAM), and any discrepancies were resolved through mutual discussion without involving a third party. A detailed data extraction template is found in supplementary Table 2.

## Risk of Bias Assessment

The methodological quality and risk of bias in the studies included were assessed using the Newcastle-Ottawa Quality Assessment Scale (NOS) [29]. This scale evaluates study quality across three dimensions: selection (up to four points), comparability (up to two points), and outcome ascertainment (up to three points). The research group discussed and determined the criteria for comparability, the length of follow-up, and the adequacy of follow-up before the quality appraisal. The primary author (ZA) and two co-authors (MMW and YAM), each with 10 articles, independently assessed the quality of the included studies. Any discrepancies in their assessments were resolved through mutual discussion. Quality was rated as high (7–9), medium (4–6), or low (0–3).

### Data Synthesis

Qualitative synthesis was used to examine the association between temporal eating patterns with CRC according to the existing studies. We presented the names and definitions of meal timing indicator variables for all included studies. Additionally, we presented the OR, HR, or RR with 95% CI of the highest versus lowest frequency for each eating frequency from the most covariate-adjusted model. We used forest plots to illustrate the association between the highest versus lowest eating frequency and the sex-specific and cancer site-specific association, if reported. Because of the wide range of highest versus lowest feeding frequency classifications and definitions of meal frequency and timing, we did not conduct a meta-analysis to assess the summary estimates quantitatively, as these would not have provided robust, interpretable findings.

## Results

### Search Results

**Fig. 1:**
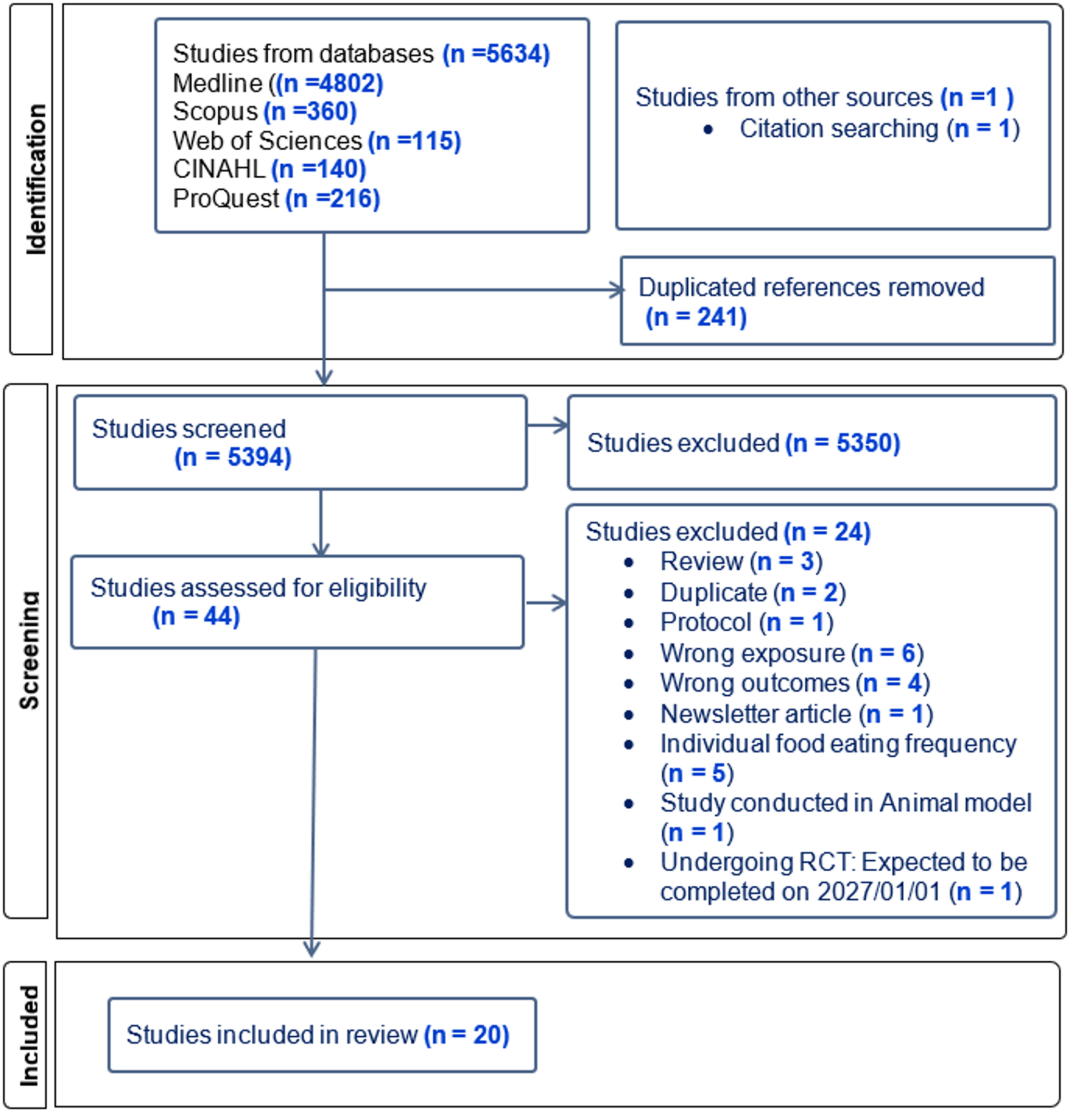
PRISMA flow chart of article search in each database

A total of 5634 records were found from the database searches. After removing 241 duplicates, 5393 articles were screened based on their titles and abstracts. Subsequently, 44 articles were screened for full text according to the inclusion criteria. From these, 20 articles were included. The search results are depicted in the PRISMA flowchart (Fig. 1).

The main reasons for excluding articles were outcomes outside the target 24-hour temporal eating pattern variables (*n* = 6), eating frequency for specific food items rather than overall diet (*n* = 5), and general cancer outcomes rather than CRC-related endpoints (*n* = 4) (Supplementary Table 3).

### Characteristics of Included Studies

**Fig. 2:**
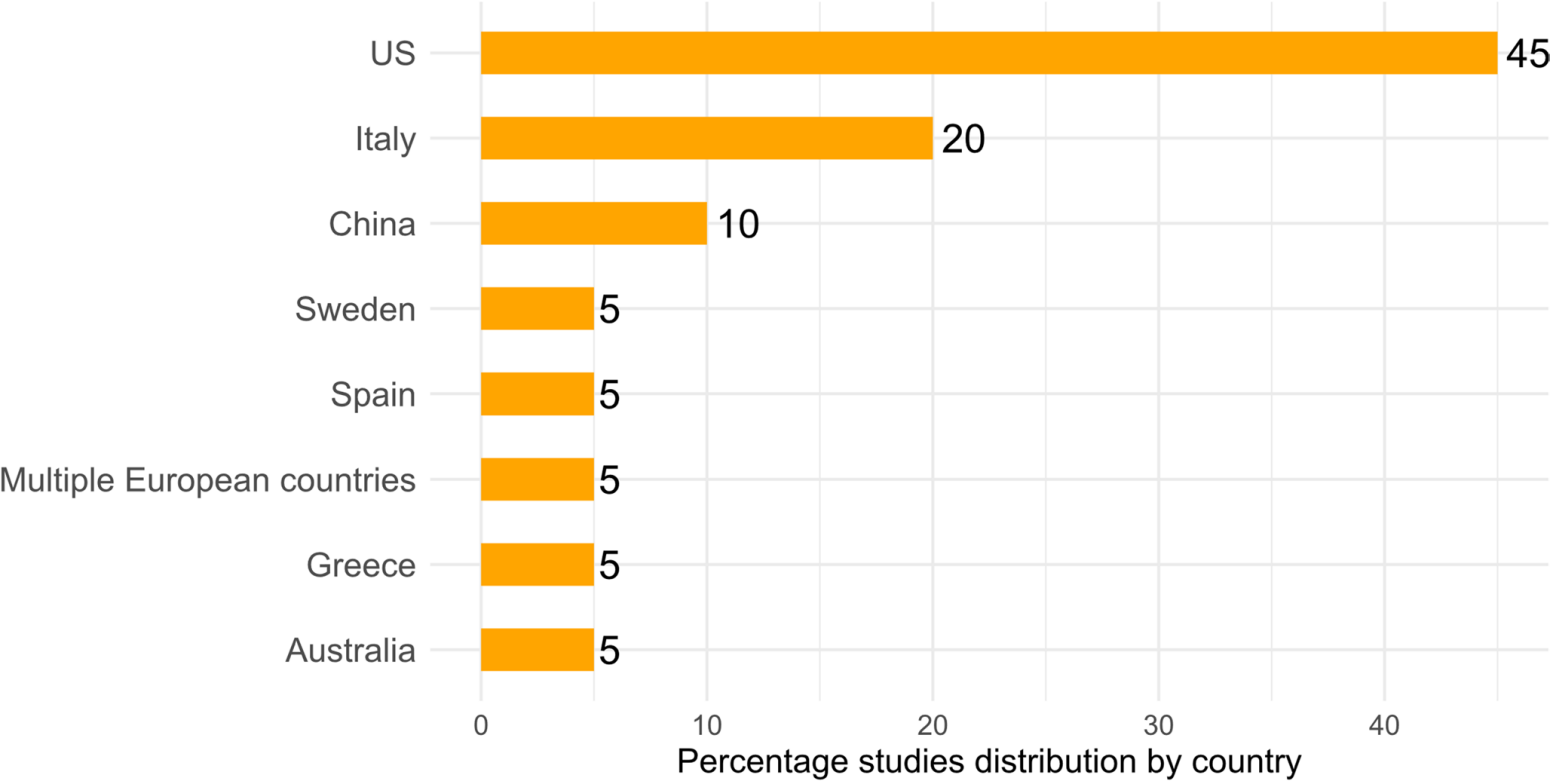
Percentage of article distribution by country. Note: Multiple European countries include Denmark, France, Germany, Greece, Italy, Netherlands, Norway, Spain, Sweden, and the United Kingdom

The characteristics of the included studies are summarised in Table 2. The review included studies published between 1986 and 2023, with sample sizes ranging from 487 to 67912 participants. Overall, data from 325,294 participants and 20 studies were included. Three studies only included female participants [30–32], and in one study only male participants [24]. Among the included studies, the majority were conducted in the US (see Fig. 2). Thirteen (65%) studies were case-control studies [22, 31, 33–43], and the remaining 35% were prospective studies [23–26, 30, 32, 44].

There was considerable heterogeneity in the utility of dietary factors. Specifically, one study considered the diet quality score, which evaluates the overall nutritional quality of an individual’s diet [25]. Meanwhile, 15 studies adjusted at least one specific food group or nutrient, highlighting the importance of particular dietary components in their analyses [22–25, 30, 35, 36, 38–40, 43, 45–47]. One study adjusted for Mediterranean dietary score [37] and another study adjusted for the dietary approaches to stop hypertension score [24]. Additionally, 12 studies focused on adjusting for energy intake [22–24, 26, 32, 36, 38, 39, 41, 45–47], which is crucial for understanding the relationship between diet and health outcomes (Table 2).

**Table 2:** Characteristics of included studies

First author and year	Country	Sample Size	Sex	Age (years)	Covariates considered during the analysis	Outcome
Prospective studies						
Bao et al., 2012 [30]	United States (US)	*N* = 55,540 (incident cases = 552)	Female	48–73	Age, smoking, BMI, physical activity, red and processed meat, and other known risk factors for CRC	CRC
Peterson et al., 2022 [23]	Multiple European countries *	*N* = 487 (mortality = 209)	Both (male, 51.3%)	59.2**	Sex, BMI, smoking status, physical activity, education, diabetes, use of NSAIDs, caloric intake per day, alcohol (grams), fibre (grams), red meat, and dairy intakes.	CRC mortality
Liu et al., 2023 [25]	China	*N* = 62,746 (incident cases = 136)	Both (male, (83.26%)	18–98	Age, sex, BMI, TC, TG, Scr, UA, smoking status, drinking status, physical activity, sedentary lifestyle, tea consumption, salt intake, high-fat diet, diabetes, occupation, family history of cancer and diet quality score	CRC
Mekary et al., 2012 [24]	US	*N* = 34,968 (incident cases = 583)	Male	40–75	Age, aspirin use, family history of CRC, previous endoscopy, use of supplements containing antioxidants, BMI, energy intake, alcohol intake, physical activity level, red meat consumption, total calcium intake, dietary folate intake, dietary vitamin D intake; pack years of smoking before 30 years of age, and Dietary Approaches to Stop Hypertension score	CRC
Perrigue et al., 2013 [26]	US	*N* = 67,912 (incident cases = 409)	Both (male, 47.55%)	50–76	Age, sex, race/ethnicity, education, BMI, MET-hours per week of moderate/vigorous activity, smoking history, energy intake, calcium intake, vitamin D intake, alcohol intake, fruit and vegetable intake, red/processed meat intake, aspirin use, non-aspirin NSAID use, family history of CRC, history of sigmoidoscopy/colonoscopy	CRC
Zhang et al., 2021 [32]	US	The total sample size and the number of deaths depend on the questions^@^	Female	20–55	Age, follow-up cycle, race, cumulative average BMI (with 4-year lag time), BMI at age 18, smoking status, time since quit smoking, pack-years of smoking, alcohol intake, physical activity, total calories intake, EDIH, regular use of aspirin, multivitamin use, menopausal status, postmenopausal hormone use, history of hypertension, history of hypercholesterolemia (with 4-year lag time), history of diabetes (with 4-year lag time), family history of cancer, and family history of cardiovascular disease.	CRC mortality
Tseng et al., 2000 [44]	US	*N* = 9978 (incident cases = 150)	Both (male, 37%)	≥ 55	Age, gender, race, energy intake, and intake of alcohol, total fat, and fibre	CRC
Case-control studies						
De Verdier 1992 [22]	Sweden	328 cases and 500 controls (*N* = 828)	Both	66–69**	Year of birth, gender, total energy, total fat, protein, dietary fibre intake	Colon cancer
Favero et al., 1998 [35]	Italy	1953 cases and 4154 controls (*n* = 6298)	Both (male, 50.78%)	< 75	Age, sex, study centre, education, physical activity, and intake of vegetables, proteins, carbohydrates, and fat	CRC
Franceschi et al., 1992 [36]	Italy	889 colon cancer cases; 581 rectal cancer cases; 2475 controls (*n* = 3945)	Both (male, 60.84%)	< 75	Age, sex, education, area of residence, BMI, approximate total energy, and red meat intake.	CRC
Lin et al., 2018 [40]	China	166 cases and 166 controls (*n* = 332)	Both (male, (62.7%))	18–75	Dinner-to-bed time, post-dinner walk, sleep duration, cigarette smoking, alcohol drinking, special dietary habits, fresh vegetable and fresh fruit intake, and family cancer history.	CRC
Shoff et al., 1997 [31]	us	126 colon and 63 rectal cancers, and 322 controls (*n* = 511)	Female	30–74	Age, previous use of sigmoidoscopy, family history of CRC, BMI, smoking status, and intake of beer, wine, and hard liquor	CRC
Wei et al., 2004 [42]	US	636 cases and 1048 controls (*n* = 1684)	Both (male, 50.42%)	40–80	Race, family history, physical activity, and total energy	Colon cancer
Benito et al., 1990 [33]	Spain	286 cases, 295 population controls, and 203 hospital controls (*n* = 784)	Both (male, 52.55%)	64.2–65^#^	Age, sex, weight 10 years prior to the interview, number of years of education, job classification, physical activity on the job, and the 6 food groups	CRC
Kontou et al., 2013 [37]	Greece	500 cases and 500 controls (*n* = 10000	Both (male, 59.00%)	63^**^	Age, sex, BMI, physical activity status, smoking habits, use of table salt, coffee intake, MedDiet Score and family history of CRC	CRC
La Vecchia et al., 1996 [39]	Italy	Cases: 828 colon cancer, 498 rectal cancer, and 2024 controls (*n* = 3350)	Both (male, 56.72%)	20–74	Age, sex and total calorie intake, beta-carotene intake, vitamin C intake, red meat intake, fat, and family history of CRC	CRC
La Vecchia et al., 1999 [38]	Italy	1225 cases and 4154 controls (*n* = 5379)	Both (male, 51.33%)	20–74	Education, area of residence, age, sex, physical activity, energy intake, vegetable intake, family history of CRC	Colon cancer
Potter et al., 1986 [41]	Australia (SA)	419 cases and 732 controls (*n* = 1151)	Both (male, 63.77%)	30–74	Total energy intake	
Coates et al., 2002 [34]	California	1969 cases and 2381 controls (*n* = 4350)	Both (male, 54.23%)	30–79	Age, family history of colorectal cancer, category of long-term vigorous leisure-time activity, BMI, use of aspirin/NSAIDs, dietary energy, g fiber/1,000 kcal, and mg calcium/1,000 kcal	Colon cancer
Young et al., 1988 [43]	US	353 cases and 618 controls (*n* = 971)	Both (male, 44.49%)	> 35	Age, sex, and 16 food items/groups	Colon cancer

*Denmark, France, Germany, Greece, Italy, Netherlands, Norway, Spain, Sweden, and the United Kingdom

^**^indicates mean age

^#^ Mean age was 64.2 years for the cases, 65.0 for population controls, and 64.2 for hospital controls

@ Eating anything at any time (sample size = 63,999 and death = 494); No concern with figure change (sample size = 65,849 and death = 495); Eating anything at any time and no concern with figure change (sample size = 40,461 and death = 283)

*CRC * colorectal cancer; *BMI *body mass index; *NSAIDs *non-steroidal anti-inflammatory drugs; *TC *total cholesterol; *TG *triglyceride; *Scr *serum creatinine; *UA *uric acid; *MET *metabolic equivalent of tasks; *EDIH *empirical dietary index for hyperinsulinemia; *MedDiet *Mediterranean diet

### Measurements of Temporal Eating Patterns

There was considerable heterogeneity in how studies operationalised 24-hour eating patterns. Two studies examined eating at any time [30, 32], and two focused on dinner to bedtime (late eating) [23, 40]. Ten studies (10%) reported on meal frequency, while five studies (10%) focused on eating occasions and five (5%) on snacking frequency. Six (30%) of the studies reported multiple 24-hour eating patterns. Studies used different criteria to assess and classify the frequency of eating per day. In ten studies, eating frequency was defined based on the participants’ classification of breakfast, lunch, dinner, and snack. Mekary et al. (2012) [24] defined meal frequency as the consumption of any food or drink, excluding coffee and tea. Coates et al. (2002) described eating frequency as the consumption of anything other than water, while Perrigue et al. (2013) defined eating frequency as the consumption of anything except coffee, tea, and soft drinks (Table 3).

None of the included studies specified a minimum calorie requirement for consideration of a meal when classifying eating opportunities. Further, none of the studies reported the duration of each meal, the time interval between each meal, meal regularity, and the time interval between the first and last meal (Supplementary Fig. 1). Except for a study conducted by Mekary et al. (2012) [24], no studies reported the energy distribution across eating opportunities in a 24-hour window.

**Table 3:** Meal timing indicator variables and definition among included studies

First author and year	Dietary assessment	Meal timing indicator variables	Exposure variable definition
Bao et al., 2012 [30]	FFQ	Meal timing	Participants were asked to answer “yes” or “no” to the question “I eat anything I want, anytime I want.”
Peterson et al., 2022 [23]	24-hour dietary recall	Eating occasions Late last eating	“Participants were asked to recall time-stamped eating episodes starting at the time of waking on the recall day until the time of waking the previous day. Meal timing variables were defined as a number of eating occasions (> 6 meals per day versus ≤ 6 meals per day), and late last eating episode (≤ or > 9 pm).”
Coates et al., 2002 [34]	Dietary history	Eating occasions Meal frequency Snack frequency	“Participants were asked to recall when they ate and drank anything other than water on a usual day during the referent year and what kinds of food or drink they typically consumed. The number of eating occasions was the total number of snacks and meals per day; thus, the possible maximum number of eating occasions was eight (3 meals and 5 snacks)”
De Verdier 1992 [22]	FFQ	Eating frequency Meal frequency Snack frequency	“Participants were asked about usual eating frequency and diet during the previous five years and were asked to exclude periods when they were affected by illness. The questionnaire ascertained the number of times per week food was eaten before breakfast, at breakfast, between breakfast and lunch, at lunch, between lunch and dinner, at dinner, and after dinner. In this study, breakfast, lunch, and dinner were considered ‘meals,’ and eating at other times was considered ‘snacking”
Favero et al., 1998 [35]	FFQ	Meal frequency	“Self-report of frequency of consumption per day and classified as ≤ 2, 3, and ≥ 4 meals per day”
Lin et al., 2018 [40]	Questionnaire on some specific dietary intake	Dinner to bedtime	Self-report of the following questions: “What time do you usually have dinner? What time do you go to bed at night?” Finally, it was classified as dinner-to-bed time (shorter: <3 hr; moderate: 3–3.9 hr; longer: ≥4 hr)
Liu et al., 2023 [25]	FFQ	Breakfast skipping	“How many days do you generally consume breakfast in a typical week?” With four alternative responses: no breakfast”, “1–2 times weekly”, “3–5 times weekly”, and “breakfast every day”
Mekary et al., 2012 [24]	FFQ	Eating frequency	“Participants were asked to report the number of times per day they ate before breakfast, at breakfast, between breakfast and lunch, at lunch, between lunch and dinner, at dinner, between dinner and bedtime, and after going to bed.” “breakfast”, “lunch”, and “dinner” were considered to be meals, whereas eating at other times was considered snacking.”
Mekary et al., 2012 [24]	FFQ	Snack frequency	“Participants could report eating from 1 to 8 times/day. Because the questionnaire focused on eating, it is most likely that drinking coffee, tea, or soda was not considered snacking in the study.”
Perrigue et al., 2013 [26]	FFQ	Eating frequency	Eating frequency was ascertained by the question ‘‘On average, how many times a day did you eat (meals plus snacks)? Snacks include food, milk, and milk beverages such as lattes. Coffee, tea, and soft drinks alone do not count as snacks’’ with defined response options of up to 7? times per day. In the following analyses, these responses were collapsed to 1–2; 3; 4; and 5 + meals or snacks per day.
Shoff et al., 1997 [31]	No information	Eating frequency	“How many meals did you eat in an average weekday two years ago?” and “In an average weekday, how many times did you eat or drink a snack two years ago? Do not count breakfast, lunch or dinner.”
Shoff et al., 1997 [31]	No information	Snack frequency	“How many meals did you eat on an average weekday two years ago?” and “On an average weekday, how many times did you eat or drink a snack two years ago? Do not count breakfast, lunch or dinner.”
Tseng et al., 2000 [45]	FFQ	Eating frequency	Interview using the following questions: “Including evening snacks, how many between-meal snacks do you have per day?” and “How many meals do you usually eat daily?” The terms “meals” and “between-meal snacks” were not further defined for participants.
Tseng et al., 2000 [45]	FFQ	Meals frequency	Interview using the following questions: “Including evening snacks, how many between-meal snacks do you have per day?” and “How many meals do you usually eat daily?” The terms “meals” and “between-meal snacks” were not further defined for participants.
Tseng et al., 2000 [45]	FFQ	Snack frequency	Interview using the following questions: “Including evening snacks, how many between-meal snacks do you have per day?” and “How many meals do you usually eat daily?” The terms “meals” and “between-meal snacks” were not further defined for participants.
Wei et al., 2004 [42]	FFQ	Eating frequency Snack frequency	Eating frequency was defined as a combination of meals and snacks. “Participants reported the number of days per week they had breakfast, lunch, and dinner over the past year. They were also asked how many times per day and how many days per week they had snacks, including all beverages, except coffee, tea, diet drinks, and water, over the past year. The number of eating episodes per day was subsequently calculated from the average of daily meals and snacks reported for a typical 1-wk period over the past year”
Zhang et al., 2021 [32]	FFQ	Meal timing	Participants answered two questions on whether “I eat anything I want, anytime I want” and “I pay a great deal of attention to changes in my figure” applied to them, with “yes” or “no” as response categories
Benito et al., 1990 [33]	FFQ	Meal frequency	No clear statement of how the participants were asked but meal frequency was classified as frequency (< 4 and ≥ 4 times/day)
Kontou et al., 2013 [37]	FFQ	Meal frequency	“Participants were asked the number of meals regularly consumed each day (measured on a quantitative scale, i.e. 1, 2, 3, 4, 5, > 5). The daily number of meals followed a monotonic scoring system, giving a score of 0 for 1 meal/d and a score of 5 for > 5 meals/d.”
La Vecchia et al., 1996 [39]	FFQ	Meal frequency	No clear statement of how the participants were asked, but meal frequency was classified as frequency < 3 and ≥ 3 times/day
La Vecchia et al., 1999 [38]	FFQ	Eating frequency	No clear statement of how the participants were asked, but eating frequency was classified as Eating frequency < 4 and ≥ 4 times/day
Potter et al., 1986 [41]	FFQ	Meal frequency	No clear statement of how the participants were asked, but eating frequency was classified as meal frequency ≤ 3, 4, and ≥ 5 times/day
Young et al., 1988 [43]	FFQ	Eating frequency	No clear statement of how the participants were asked, but eating frequency was classified as Eating frequency ≤ 5 and ≤2 per day

Key: *FFQ *food frequency questionnaire

### Meal Frequency and CRC

In several case-control studies, higher eating frequency was associated with an increased risk of CRC (Table 4). For instance, in a study with 328 cases and 500 controls, participants who ate more than six times per day had an odds ratio (OR) of 2.0 (95% CI: 1.0, 3.9), and those who ate five to six times per day had an OR of 2.0 (95% CI: 1.2, 3.5) compared to those who ate three times per day [22]. Similarly, a study by Favero et al. (1998) found that individuals who ate more than four times a day had an increased risk of CRC (OR = 1.24, 95% CI: 1.0, 1.54) compared to those who ate twice or fewer times per day [35]. Another study by Franceschi et al. (1992), with 889 cases and 4275 controls, found that individuals who ate four or more times per day had an OR of 1.9 (95% CI: 1.1, 3.3), and those who ate three times per day had an OR of 1.7 (95% CI: 1.5, 2.1), indicating an increased risk of colon cancer [36] relative to those who ate two or fewer times per day.

**Fig. 3:**
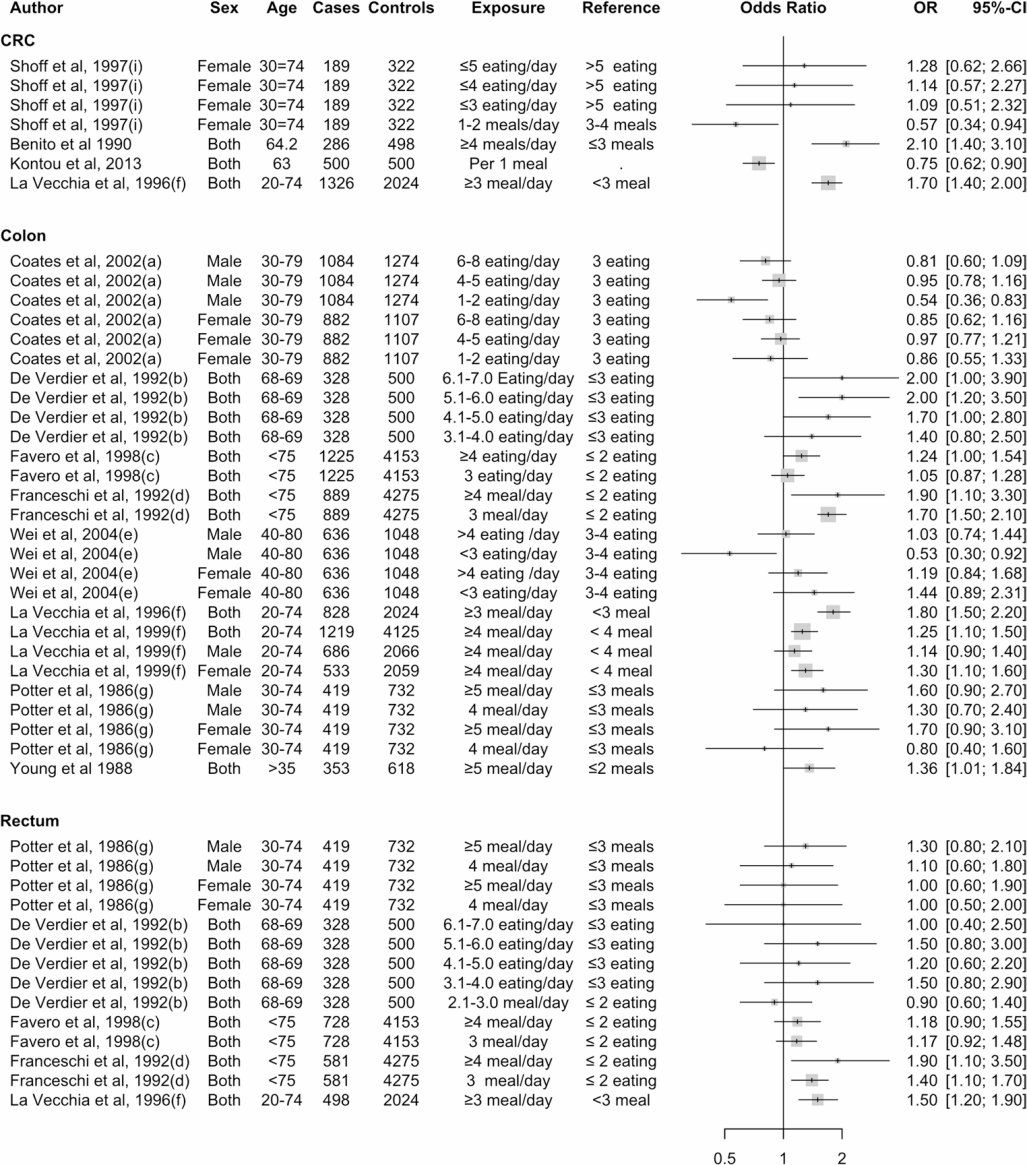
Summary of the associations reported in the literature between meal frequency and CRC from case-control studies. Note: The letter in brackets following the author and year indicates 24-hour eating patterns extracted from the same study

Two case-control studies found that eating less frequently was linked to a reduced risk of CRC in men; however, this was not observed in women. Coates et al. (2002) [34] and Wei et al. (2004) [42] found that men who ate less than three times a day had 46% (OR = 0.54; 95% CI: 0.36, 0.86) and 47% (OR = 0.53; 95% CI: 0.30, 0.92) lower odds of CRC compared to those who ate three meals per day (Fig. 3).

**Fig. 4:**
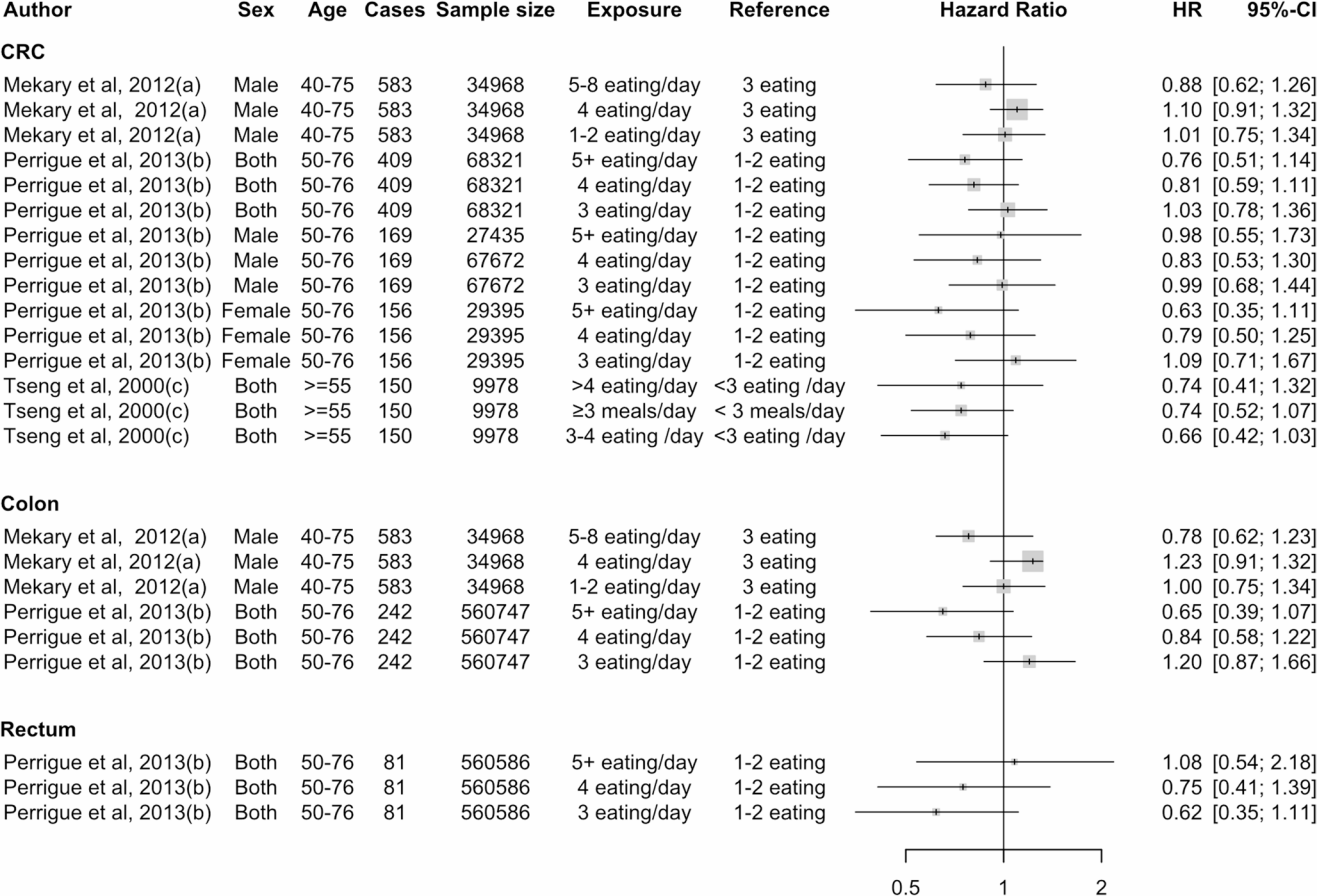
Summary of the association between meal frequency and CRC from prospective studies. Note: The letter in brackets following the author and year indicates 24-hour eating patterns extracted from the same study

In contrast to case-control studies, evidence from prospective studies suggests that a higher eating frequency may decrease the risk of CRC. However, these studies does not point to a clear association between eating frequency and CRC (Fig. 4). Asdepicted in Fig. 3, exposure and reference variables used to measure the association between meal frequency and CRC risk are highly variable, making interpretation of these relationships challenging in the existing literature.

**Fig. 5:**
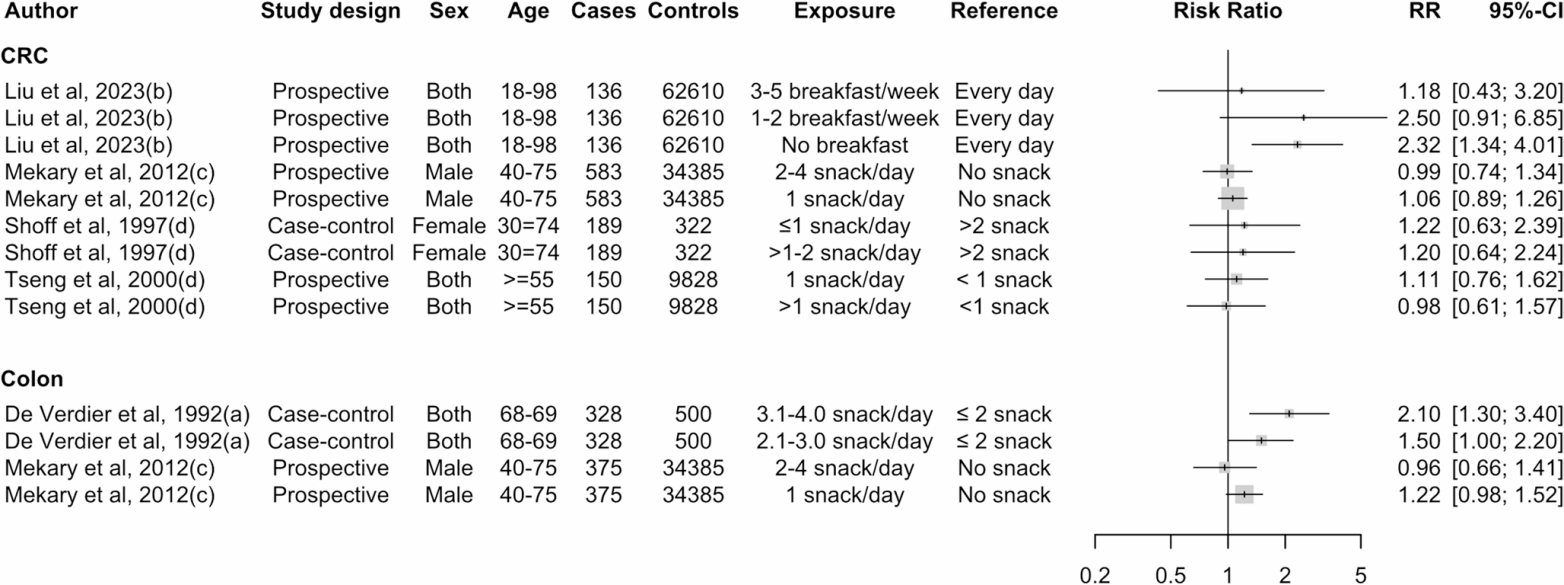
Snack and breakfast frequency and risk of CRC. Note: The letter in brackets following the author and year indicates 24-hour eating patterns extracted from the same study

The relationship between snack frequency and CRC was not clear from the existing literature (Fig. 5). One study [22] showed that consuming more than two snacks per day is associated with CRC (RR = 2.10; 95% CI: 1.30, 3.40), while another study by Shoff et al. (1997) found a non-significant association with CRC [31]. Compared to taking breakfast every day, those who had no history of breakfast were associated with a 32% increased risk of CRC (HR = 2.32; 95% CI: 1.34, 4.01) compared to having breakfast every day [24].

### Timing of Eating and CRC

**Fig. 6:**
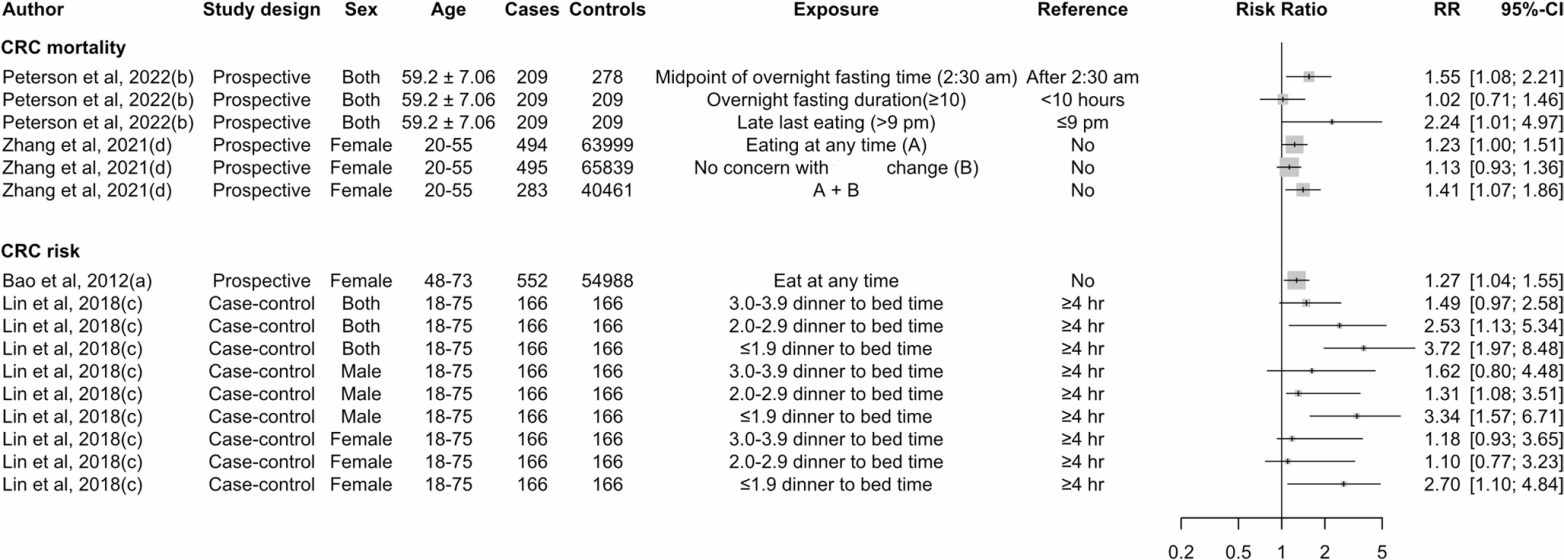
Summary of the association between meal timing and CRC risk and mortality. Note: A + B in Zhang et al. 's study represents two questions asked to respondents. The questions were: "Do I eat anything I want, anytime I want?" and "Do I pay a great deal of attention to changes in my figure?" Respondents answered with"yes" or "no" for each question. The letter in brackets following the author and year indicates 24-hour eating patterns extracted from the same study. The measure of association for Lin et al. 2018 is OR

Two studies have investigated the relationship between eating behaviours and the risk of CRC, focusing on different aspects of meal timing. A case-control study by Lin et al. (2018) found that a shorter time interval between dinner and bedtime was positively correlated with the risk of CRC [40]. Specifically, a time interval of less than 2 hr between dinner and bedtime was associated with higher CRC risk (HR = 3.72; 95% CI: 1.97, 8.48), with the association being particularly strong among males (HR = 3.34; 95% CI: 1.58, 6.71). A study conducted by Bao et al. (2012) more broadly demonstrated that eating at any time, regardless of proximity to bedtime, was associated with an elevated CRC risk (HR = 1.27; 95% CI: 1.04–1.55) [30] (Fig. 6).

### Meal Frequency, Timing, and CRC Mortality

Two studies examined the association between 24-hour temporal eating patterns and CRC mortality. The study conducted by Peterson et al. (2022) assessed both meal frequency and meal timing [23], while another study by Zhang et al. (2021) [32] focused solely on meal timing. The finding of Peterson et al. (2022) showed that individuals who had six eating occasions per day did not have an increased risk of CRC mortality (HR = 1.11; 95% CI: 0.75, 1.65) compared to those with fewer than six eating occasions per day [23]. However, the study indicated that eating episodes after 9 PM were associated with an increased risk of CRC mortality (HR = 2.24; 95% CI: 1.01, 4.97). Similarly, the study by Zhang et al. (2021) [32] found a 23% increased risk of CRC mortality (HR = 1.23; 95% CI: 1.00, 1.51) (Fig. 6).

**Table 4:** Summary of association of temporal meal pattern and CRC risk and mortality

Study	Direction of association	Additional comments
***Exposure***: *Higher eating frequency* ***Outcome***: *CRC risk*		
Coates et al. 2002 [48]	**↓**	The point estimates showed a reduced risk
De Verdier et al., 1992 [22]	**↑**	The pattern showed an increased risk
Favero et al. 1998 [35]	**↑**	The pattern showed an increased risk
Franceschi et al., 1992 [36]	**↑**	The pattern showed an increased risk
Wei et al. 2004 [47]	**↑**	Less than three meals a day was protective for males but increased the risk for females
La Vecchia et al. 1996 [39]	**↑**	Showed consistent risk association with colon, rectum and aged less than 60 years and greater than 60 years
La Vecchia et al., 1999 [38]	**↑**	Increased risk only for females, but not males
Potter et al. 1986 [41]	**↑**	The point estimates showed an increased risk
Young et al. 1988 [43]	**↑**	Increase risk only for those aged > 35 years
Shoff et al. 1997 [31]	**↓**	The point estimates for lower eating frequency showed an increased risk
Benito et al. 1990 [33]	**↑**	The finding showed a statistically significant association
Kontou et al. 2013 [37]	**↓**	The risk of CRC decreased by 0.75 when eating frequency increased by one per day
Mekary et al. 2012 [24]	**↓**	The point estimates showed a decreased risk
Perrigue et al. 2013 [26]	**↓**	The point estimates showed a decreased risk. However, the point estimates showed an increased risk for rectum cancer
Tseng et al. 2000 [45]	**↓**	The point estimates showed a decreased risk
***Exposure***: *Shorter time interval between last meal and bedtime*,* snacking frequency and skipping breakfast* ***Outcome***: *CRC risk*		
Lin et al. 2018 [40]	**↑**	Increased risk of CRC was observed across patterns of time interval
Bao et al. 2012 [30]	**↑**	Eating anything at any time increases the risk
Liu et al. 2023 [25]	**↑**	The pattern showed an increased risk across the frequency of breakfast intake per week
Mekary et al. 2012 [24]	**ࣧ**	The pattern doesn’t show decreased association across each snacking frequency
De Verdier et al. 1992 [22]	**↑**	The pattern showed an increased risk across the frequency of snacking
Shoff et al. 1997 [31]	**↓**	The point estimate showed that less than one snack per day increases CRC risk
Tseng et al. 2000 [45]	**ࣧ**	The pattern doesn’t show decreased association across each snaking frequency
Peterson et al. 2022 [23]	**↑**	Eating after 9 pm increases the risk of mortality
Zhang et al. 2021 [32]	**↑**	Eating at any time increases the risk

↓ indicates decreased risk; ↑ indicates increased risk; — indicates no association

### Risk of Bias

All prospective studies included were rated as high quality. Of 13 case-control studies, more than half (7 studies, 53.8% were rated as medium quality (Table 5).

**Table 5:** The Newcastle-Ottawa Scale (NOS) quality appraisal for included studies^#^

Author, year	Selection				Comparability		Outcome^@^			Score	Quality
	1	2	3	4	1 A	1B	1	2	3		
Prospective studies											
Bao et al. 2012 [30]	*	*		*	*	*	*	*	*	8	High
Peterson et al. 2022 [23]	*	*	*	*		*	*	*	*	8	High
Liu et al. 2023 [25]	*	*		*	*	*	*		*	7	High
Mekary et al. 2012 [24]	*	*		*	*	*	*	*	*	8	High
Perrigue et al. 2013 [26]	*	*		*	*	*	*		*	7	High
Zhang et al. 2021 [32]	*	*		*	*	*	*	*	*	8	High
Tseng et al. 2000 [45]	*	*		*		*	*	*	*	7	High
Case-control studies											
De Verdier. 1992 [22]	*	*	*			*		*	*	6	Medium
Favero et al. 1998 [35]	*	*		*		*	*	*	*	7	High
Franceschi et al. 1992 [36]	*	*		*		*	*	*	*	7	High
Lin et al. 2018 [40]	*	*		*	*	*		*	*	7	High
Shoff et al. 1997 [31]	*	*	*	*	*	*		*	*	8	High
Wei et al. 2004 [47]	*	*	*			*		*	*	6	Medium
Benito et al. 1990 [33]	*	*	*					*	*	5	Medium
Kontou et al. 2013 [37]	*	*	*	*	*	*		*		7	High
La Vecchia et al. 1996 [39]	*	*		*	*	*		*	*	7	High
La Vecchia et al. 1999 [38]	*	*		*	*	*			*	6	Medium
Potter et al. 1986 [41]	*	*	*			*			*	5	Medium
Coates et al. 2002 [34]	*	*	*		*	*		*		6	Medium
Young et al. 1988 [43]	*	*	*						*	4	Medium

@For case-control studies, the outcome became exposure

# For prospective studies each item included the following domains: Selection (1–4 stars): representativeness of the exposed cohort, selection of the non-exposed cohort, ascertainment of exposure, demonstration that outcome of interest was not present at the start of the study; Comparability (1–2 stars): The study should be adjusted for age, sex and family history of CRC, and the study additionally adjusted for one of the following body mass index (BMI), smoking, alcohol drinking, energy intake; Outcome (1–3 stars): Assessment of outcome, a 10 and 5-year follow-up long enough for CRC cancer risk and mortality, respectively to occur, a 80% follow-up of cohorts at the end of the study period

#For case-control studies each item included the following domains: Selection (1–4 stars): adequate case definition of cases, representative of cases, community control, no history of CRC among controls; Comparability: The study should be adjusted for age, sex and family history of CRC, and the study additionally adjusted for one of the following body mass index (BMI), smoking, alcohol drinking, energy intake; Exposure (1–3 stars): structured interview where blind to case/control status for ascertainment of exposure, the same methods of ascertainment for cases and control, and the same non-response rate for cases and control

*The star represents the study fulfilling the respective NOS quality appraisal criteria, and those not fulfilling the criteria have not been awarded a star

## Discussion

This systematic review aimed to explore the association between 24-hour temporal eating patterns with CRC risk and mortality. Highly heterogeneous measures of food intake and 24-hour temporal eating pattern indicators prevented meaningful meta-analysis and made it challenging to synthesise the existing literature. Findings from the case-control studies suggest that a higher frequency of eating occasions might be associated with elevated risk of CRC. However, prospective studies did not confirm this association. The findings also suggest that skipping breakfast and a higher frequency of snacking might increase the risk of CRC. Late-night eating was associated with increased CRC risk and mortality. However, the included studies lack standard definitions and classifications of meal frequency and timing, which must be addressed in future studies to best understand associations with CRC risk and mortality.

Higher eating frequency was associated with CRC risk in case-control studies, while prospective studies indicated an inverse relationship. This could be partly explained by the fact that case-control studies are prone to recall biases, as patients may change their feeding frequency and meal timing due to disease or in response to treatment [48]. Additionally, in prospective studies, eating frequency was measured only once at baseline, which may not reflect the usual eating pattern, and participants may change dietary their behaviour during the follow-up period [49]. These methodological challenges could affect estimates of the association between feeding behaviour and CRC risk.

A key challenge in understanding these associations is the lack of a standardised definition for eating frequency and timing. Most studies relied on participants’ self-reported eating habits, with varying reference periods. Some measured eating occasions, including meals, snacks, and breakfasts, while others considered only main meals. Recall periods ranged from one day to two years, contributing to inconsistent findings and almost certainly contributing to the mixed findings in the existing literature. In the last decade, reliable and valid food timing tools have emerged, along with a measurable definition for eating frequency, offering opportunities to address these limitations [50]. For example, the American Heart Association defines eating frequency as any food or drink consumption totalling more than 210 KJ (50.19 kcal) within a 15-minute time frame [50]. Applications of standardised dietary measures in future will assist with improving this variability. Finally, it might be explained by the types of food consumed. A higher eating frequency of healthy foods, such as fruits, vegetables, and whole grains, might potentially decrease the risk of CRC [51]. In contrast, a diet that includes a high intake of unhealthy foods such as processed snacks, sugary beverages, and red or processed meats can elevate the risk of developing CRC [52–54].

Few studies examined the relationship between breakfast or snacking frequency and CRC risk. These studies found no significant associations, and there are no previous systematic reviews or meta-analyses on the topic. However, a meta-analysis of observational studies has shown that skipping breakfast was associated with increased overall cancer mortality [55], potentially due to its contribution to overweight and obesity [56].

While this review did not provide a pooled estimate of temporal eating patterns and CRC outcomes by biological sex, the findings suggest a slightly stronger association for males. The HR from prospective studies for males ranged from 0.81 to 1.23 when comparing those with higher eating frequencies to their counterparts. In contrast, the RR for females ranged from 0.66 to 1.09. Additionally, the HR for males ranged from 1.31 to 3.34, while for females it ranged from 1.10 to 2.74 when comparing participants based on the time interval between their last meal and bedtime. This suggests that temporal eating patterns may have a strong association with male sex. This statement is further supported by previous research that highlights notable gender differences in eating behaviours. Women generally eat more frequently, report higher morning hunger [57], and consume healthier foods [58, 59], while men often skip snacks, experience evening hunger, and exhibit irregular eating habits [60, 61]. This suggests that eating patterns and behaviours could significantly influence CRC risk differently based on sex, supporting the need for tailored dietary recommendations in preventive health strategies.

The association between temporal 24-hour eating patterns and CRC has not been extensively studied. However, given the robust findings between 24-hour temporal eating patterns and other health outcomes [62–64], it is clear that more research is needed to explore whether similar patterns exist for CRC. There is presently great variation in the definition of eating occasions. This makes it challenging to compare and contrast existing findings, as researchers have used various measurements for meal frequency (as described in Table 3). Moving forward, it is important to establish a standardised definition, and corresponding measurement approaches, to improve our understanding of temporal eating patterns and CRC risk. One suggested definition, as outlined by the American Heart Association, is to classify a meal as a contribution of at least 50 kcal occurring 15 min apart [50]. Additionally, it will be important to set a clear definition for meals and snacks based on their contribution to total energy intake (TEI). Meals are defined as eating occasions that provide at least 15% of TEI, while anything below that threshold is considered a snack, irrespective of the time of day or the type of food or beverage consumed [50]. Furthermore, incorporating a diet quality indicator, along with an assessment of temporal eating patterns, is vital for a comprehensive understanding. This may pose challenges for researchers in terms of measurement at scale, which must be addressed – potentially with electronic tools [65]. Understanding the potential impact of meal frequency and timing on CRC risk could provide valuable insights for prevention strategies and dietary guidelines aimed at reducing the incidence of CRC.

### Biological Mechanisms between Timing and Frequency of Eating with CRC

**Fig. 7:**
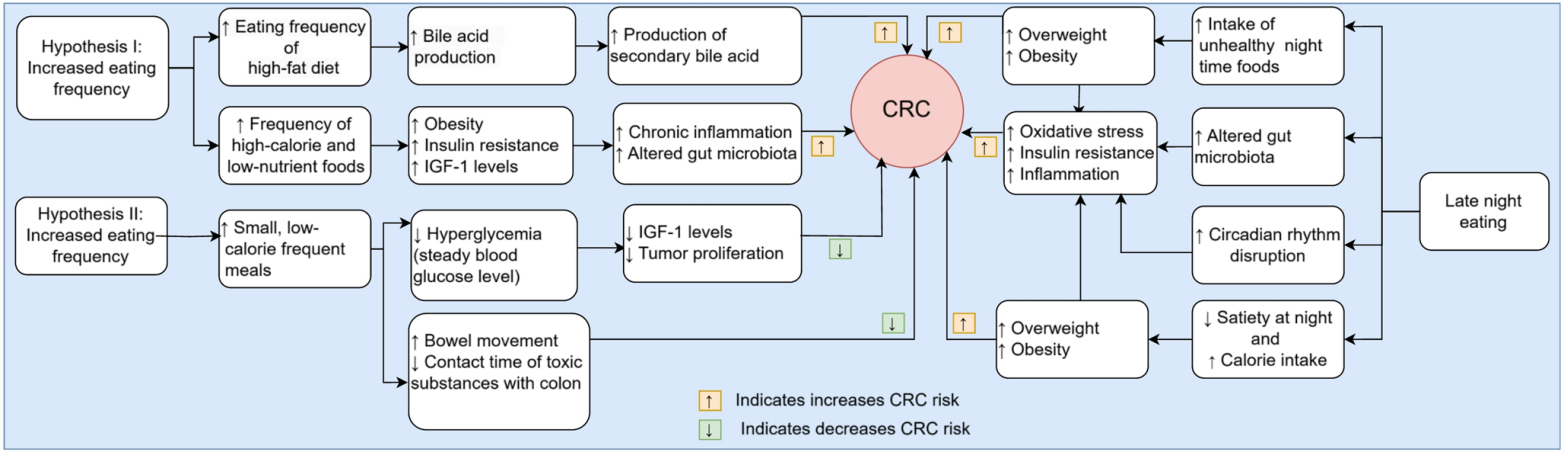
A conceptual summary of the plausible biological mechanisms between eating frequency and late-night eating with CRC. This figure illustrates two contrasting hypotheses on how eating frequency and late-night eating may influence CRC risk through metabolic and physiological pathways. The green downward arrow indicates a decrease in risk, while the orange upward arrow indicates an increase in risk. Keys: CRC = colorectal cancer, IGF-1 = insulin like growth factor

The mechanisms through which meal timing and frequency affect the risk of CRC have been summarised in Fig. 7. The relationship between eating frequency and CRC risk is explained through two main hypotheses. The first hypothesis suggests that higher eating frequency may increase CRC risk. Frequent eating, particularly in high-fat diets, stimulates bile acid production and recirculation, leading to the formation of secondary bile acids [52–54] that can damage colorectal DNA and promote carcinogenesis [66]. Additionally, frequent consumption of high-calorie, nutrient-poor foods may contribute to CRC risk by promoting obesity, elevating insulin and insulin-like growth factor-1 (IGF-1) levels, altering the gut microbiota, and increasing chronic inflammation, all of which foster a pro-carcinogenic environment [67]. Conversely, the second hypothesis suggests that higher eating frequency could reduce CRC risk. Eating smaller, more frequent, low-calorie meals helps stabilise blood glucose levels, reducing hyperglycemia and hyperinsulinemia [51], which in turn lowers IGF-1 production, which is a known CRC risk factor [68]. Additionally, frequent small meals may also promote regular bowel movements, minimising the colon lining’s exposure to potential dietary carcinogens [69].

Late-night eating could be associated with an increased CRC risk through several mechanisms. It can disrupt the body’s circadian rhythms, regulated by the brain’s suprachiasmatic nuclei and peripheral clocks, contributing to circadian disruptions that are recognised as CRC risk factors [70]. While cancer prevention guidelines emphasise reducing red and processed meats and increasing fruits, vegetables, and whole grains, they do not currently address meal timing [12]. Moreover, late-night meals are often high in sugar, fat, and refined grains [71], which contribute to oxidative stress, insulin resistance, and inflammation, all linked to CRC development [72, 73].

Late-night eaters also tend to skip breakfast [74], a behaviour associated with increased gastrointestinal cancer risk [25] and cancer-related mortality [55, 75]. Additionally, metabolic disruptions during nighttime are associated with high energy intake during the night [76]. Nighttime meals are less satiating, leading to higher calorie intake and overeating [77], especially when coupled with inactivity, further increases CRC risk [78]. Consuming large meals late at night, which disproportionately contributes to daily energy intake, exacerbates metabolic strain and elevates risks such as obesity, hypertension, and hyperglycemia, collectively enhancing CRC risk [79–81].

### Implications

The importance of the quality and amount of food is mostly cited in dietary guidelines, such as the WCRF, in order to prevent CRC [12]. However, these guidelines only focus on the amount and types of food consumed to prevent CRC. They do not take into consideration factors such as meal timing, including how often food is consumed, duration of eating window, the distribution of daily energy intake, the duration of each meal, the timing of late meals, and meal regularity. It is important to note that temporal feeding patterns have an impact on various health outcomes such as obesity, hypertension, cardiovascular disease, sleep, and overall mortality [15, 82–84]. Additionally, the number of meals consumed in a day is linked to the quality of the diet [85]. Therefore, it is crucial to comprehensively assess temporal 24-hour eating behaviour to provide recommendations that address not only the types and amounts of food but also the timing and frequency of meals in dietary guidelines.

### Strengths and Limitations

This review was conducted based on an a priori-developed protocol and comprehensive search strategies. Additionally, the review comprehensively assessed the association between 24-hour temporal eating patterns and CRC. However, it has the following limitations. First, since the included studies were conducted with different definitions of meals, estimate measures, and variability in classifying low and high eating frequency as well as meal timing, meta-analysis was not possible to examine the pooled effect of meal frequency, skipping breakfast, snacking frequency and late last mealtime on CRC risk. Secondly, none of the included studies clearly define what constitutes an eating occasion (main meal or snack). This lack of clarity may lead to under or over-reporting of eating frequency, thereby affecting the conclusion on the association between eating frequency and the risk of CRC. The included studies defined meals and snacks based on participant identification, such as lunch, dinner, breakfast, snack, and supper, and based on the time-of-day report, including morning tea, afternoon tea, or beverages, respectively [86]. In future studies, it might be helpful to adopt a standard definition of a meal based on the percentage of energy contributed to total energy intake. If the contribution is less than 15% of the total energy intake, it should be classified as a snack, regardless of the time of day. This approach may enhance the understanding of 24-hour eating patterns and their association with CRC risk [50].

## Conclusion

The findings of this review highlighted that a short interval between the last meal and bedtime increases the risk of CRC, likely due to factors like circadian rhythm disruption, oxidative stress, and inflammation. Moreover, increased meal frequency, especially with an unhealthy diet, may also raise CRC risk. The relationship between the other 24-hour eating pattern components and CRC risk remains unexplored. Comprehensive research on the association of 24-hour temporal eating patterns with CRC is limited, highlighting the need for further investigation. Future prospective or clinical trial studies are needed using standardised definitions and measurements of meal timing and frequency. Including variables such as energy distribution across meals, meal timing duration, intervals between meals, the time interval from the first to the last eating episode, meal regularity, and the interval between the last meal and bedtime could provide an additional advantage in examining the effect of 24-hour temporal eating pattern on CRC and contribute to overall cancer prevention strategies.

## Key References

- Peterson LM. Investigating Meal Timing in Colorectal Cancer Survivorship. United States -- Utah: The University of Utah; 2022.
  - This article comprehensively assessed the effect of meal timing on CRC mortality with relatively to a relatively longer follow-up, 17 years. The article specifically examined the midpoint of overnight fasting and late-night eating as well as meal frequency with possible mechanisms.
- Liu T, Wang Y, Wang X, Liu C, Zhang Q, Song M, et al. Habitually skipping breakfast is associated with the risk of gastrointestinal cancers: evidence from the Kailuan cohort study. Journal of General Internal Medicine. 2023;38(11):2527-36. doi: 10.1007/s11606-023-08094-7. Epub 2023 Mar 3.
  - The study prospectively examined the effects of breakfast frequency on the occurrence of GI including a relatively larger sample size with extensive statistical analysis
- Zhang Y, Song M, Yuan C, Chan AT, Schernhammer ES, Wolpin BM, et al. Unrestrained eating behaviour and risk of mortality: A prospective cohort study. Clinical Nutrition. 2021;40(11):5419-29.
  - The article prospectively examined the effect of meal timing on CRC with a relatively larger sample and longer follow-up period.

## Electronic Supplementary Material

Supplementary Material 1. (368 KB)

## Data Availability

No datasets were generated or analysed during the current study.
