# Supplementary material for: Temporal Eating Patterns and Colorectal Cancer: A Systematic Review
Source: Curr Nutr Rep. 2025 Oct 21;14(1):114. doi: 10.1007/s13668-025-00700-w (PMC12540529; doi:10.1007/s13668-025-00700-w)
Supplement: Supplementary file 1 — Supplementary Material 1. (368 KB) [file 13668_2025_700_MOESM1_ESM.docx]

**Temporal eating patterns and colorectal cancer: a systematic review**

First author: Zegeye Abebe^1,2^

^1^Flinders Health and Medical Research Institute, College of Medicine and Public Health, Flinders University, Adelaide, South Australia, 5042

^2^Department of Human Nutrition, Institute of Public Health, College of Medicine and

**Online Supplementary Materials**

**Supplemental Table legends:**

**Supplementary Table 1:** Search strategy in Ovid Medline and master search

**Supplementary Table 2**: Summary of data extraction from the eligible articles

**Supplementary Table 3**: Reason for exclusion of articles

**Supplemental Figure legends:**

Figure 1: Studies that examined temporal eating patterns with CRC risk or mortality

**Supplementary Table 1:** Search strategy in Ovid Medline and master search

| # | Medline | Master search |
| --- | --- | --- |
| 1 | exp neoplasms/ | exp neoplasms/ |
| 2 | ((Cancer or Neoplasms or Digestive system or Gastrointestinal or Esophageal or Esophageal or Squamous Cell or Intestin* or Cecal or Appendiceal or Appendix or Colorectal or Colon* or Colitis-Associated or Sigmoid or Rectal or Rectum or Anal or Anus or Duodenal or Ileal or Gastric or Hepto* or Heptic* or Hepta* or Liver or Pancreatic or Islet Cell or Stomach or bowel) adj3 (cancer* or neoplas* or tumour or tumor or carcinoma or metast* or Adenoma or adenocarcinoma)).tw,kf. | ((Cancer OR Neoplasms OR "Digestive system" OR Gastrointestinal OR Esophageal OR Esophageal OR "Squamous Cell" OR Intestin* OR Cecal OR Appendiceal OR Appendix OR Colorectal OR Colon* OR "Colitis-Associated" OR Sigmoid OR Rectal OR Rectum OR Anal OR Anus OR Duodenal OR Ileal OR Gastric OR Hepto* OR Heptic* OR Hepta* OR Liver OR Pancreatic OR Islet Cell OR Stomach OR bowel) adj3 (cancer* OR neoplas* OR tumour OR tumor OR carcinoma OR metast* OR Adenoma OR adenocarcinoma)) |
| 3 | (Adenomatous Polyposis Coli or Gardner Syndrome).tw,kf. | (Adenomatous Polyposis Coli OR Gardner Syndrome) |
| 4 | or/1-3 | 1 OR 2 OR 3 |
| 5 | Eating/ or Feeding Behavior/ | Eating/ OR Feeding Behavior/ |
| 6 | exp Meals/ | exp Meals/ |
| 7 | exp Diet/ | exp Diet/ |
| 8 | 5 or 6 or 7 | 5 OR 6 OR 7 |
| 9 | Diet, Healthy/ | Diet, Healthy/ |
| 10 | Diet, Western/ | Diet, Western/ |
| 11 | Feeding Behavior/ | Feeding Behavior/ |
| 12 | ("Eating window" or "eating interval" or "Mistimed eating" or "Dinner to bedtime" or "Eating frequency" or "Eating occasions" or "post-dinner and sleep" or "Time gap between each meal" or "Time-restricted eating" or "Late eating" or "Meal timing" or "Eating duration" or "Late night dinner" or "Eating habits, diet" or "feeding behavior" or meal or diet).tw,kf. | ("Eating window" OR "eating interval" OR "Mistimed eating" OR "Dinner to bedtime" OR "Eating frequency" OR "Eating occasions" OR "post-dinner and sleep" OR "Time gap between each meal" OR "Time-restricted eating" OR "Late eating" OR "Meal timing" OR "Eating duration" OR "Late night dinner" OR "Eating habits, diet" OR "feeding behavior" OR meal or diet).tw,kf. |
| 13 | 8 or 9 | 8 OR 9 |
| 14 | 4 and 10 | 4 AND 10 |

**Supplementary Table 2**: Summary of data extraction from the eligible articles

| **Domains** | **Data to be extracted** |
| --- | --- |
| General | Name of the first author  Year of publication  CRC risk or mortality |
| Methods | Study area/country  Study design  Study participants  24-hour eating pattern indicator (meal frequency, meal timing, snacking frequency, breakfast etc)  The measure of the association between 24-hour eating pattern indicator  Tools used for measuring 24-hour eating pattern indicator  CRC risk or mortality assessment methods |
| Results | Cases and the total number of participants  Age group of study participants when applicable  Sex  Estimates of association with 95%CI  24-hour eating pattern indicator  Main conclusion |

**Supplementary Table 3**: Reason for exclusion of articles

| Authors | Title | Reason for exclusion |
| --- | --- | --- |
| Mirghani et al, 2019 | The relationship between sleep duration, dinner to bedtime, and colorectal cancer: a review of the literature | Review |
| Wei et al, 2003 | Eating frequency and colon cancer risk | Duplicate |
| Liu et al, 2020 | Study on the relationship between diet and environmental exposure-related factors and the incidence of colorectal cancer | Individual food eating frequency |
| Gabel et al, 2022 | The basis and design for time-restricted eating compared with daily calorie restriction for weight loss and colorectal cancer risk reduction trial (TRE-CRC trial) | Protocol |
| Jin et al, 2024 | Epidemiological characteristics of early-onset colorectal cancer: a prospective cohort study from a single center | Wrong exposure |
| De Verdier et al, 1992 | Eating frequency-a neglected risk factor for colon cancer? | Duplicate |
| Nadeem et al, 2023 | Dietary risk factors in gastrointestinal cancers: A case-control study in North India. | Wrong exposure |
| Collatuzzo et al, 2022 | Consumption of Yoghurt and Other Dairy Products and Risk of Colorectal Cancer in Iran: The IROPICAN Study. | Wrong exposure |
| Bishehsari et al, 2020 | Abnormal Eating Patterns Cause Circadian Disruption and Promote Alcohol-Associated Colon Carcinogenesis. | The study was conducted in an animal model |
| Vernia et al 2021 | Dietary Factors Modulating Colorectal Carcinogenesis. | Review |
| Ganjavi et al, 2019 | Late effect of the food consumption on colorectal cancer rate. | Wrong exposure |
| Alsheridah et al, 2018 | Diet, obesity and colorectal carcinoma risk: results from a national cancer registry-based middle-eastern study. | Individual food eating frequency |
| Mafiana et al, 2018 | Association between Dietary and Lifestyle Indices and Colorectal Cancer in Oman: A Case-Control Study. | Individual food eating frequency |
| Zhang et al, 2018 | The joint effects of major lifestyle factors on colorectal cancer risk among Chinese men: A prospective cohort study. | Wrong exposure |
| Lewis et al, 2016 | Racial differences in dietary changes and quality of life after a colorectal cancer diagnosis: a follow-up of the Study of Outcomes in Colorectal Cancer Survivors cohort. | Wrong outcomes |
| Saetang et al, 2017 | Diets link metabolic syndrome and colorectal cancer development (Review). | Exclusion reason: Review; |
| Angelo et al, 2016 | Dietary risk factors for colorectal cancer in Brazil: a case control study. | Individual food eating frequency |
| Zhong et al, 2014 | Dietary fiber and fiber fraction intakes and colorectal cancer risk in Chinese adults. | Wrong exposure |
| Mahfouz et al, 2014 | The role of dietary and lifestyle factors in the development of colorectal cancer: case control study in Minia, Egypt. | Individual food eating frequency |
| Robinson et al, 1993 | Is dietary restriction always necessary in Haemoccult screening for colorectal neoplasia?. | Wrong outcomes |
| Tsiountsioura et al, 2014 | Detailed assessment of nutritional status and eating patterns in children with gastrointestinal diseases attending an outpatients clinic and contemporary healthy controls. | Wrong outcomes |
| Translated by Content Engine LLC, 2023 | Eating habits that can lead to colon cancer | Newsletter article |
| Li 2023 | Clinical Trial: Time-Restricted Eating Versus Nutritional Counseling for the Reduction of Radiation or Chemoradiation Tx Side Effects in Patients With Prostate, Cervical, or Rectal Cancers | Undergoing RCT: Expected to be completed on 1027/01/01; |
| Huang et al, 2001 | Eating frequency, intake of flavonoids, glutathione and trypsin inhibitors, use of nonsteroidal anti -inflammatory drugs and the prevalence of colorectal adenomas | Colorectal adenoma |


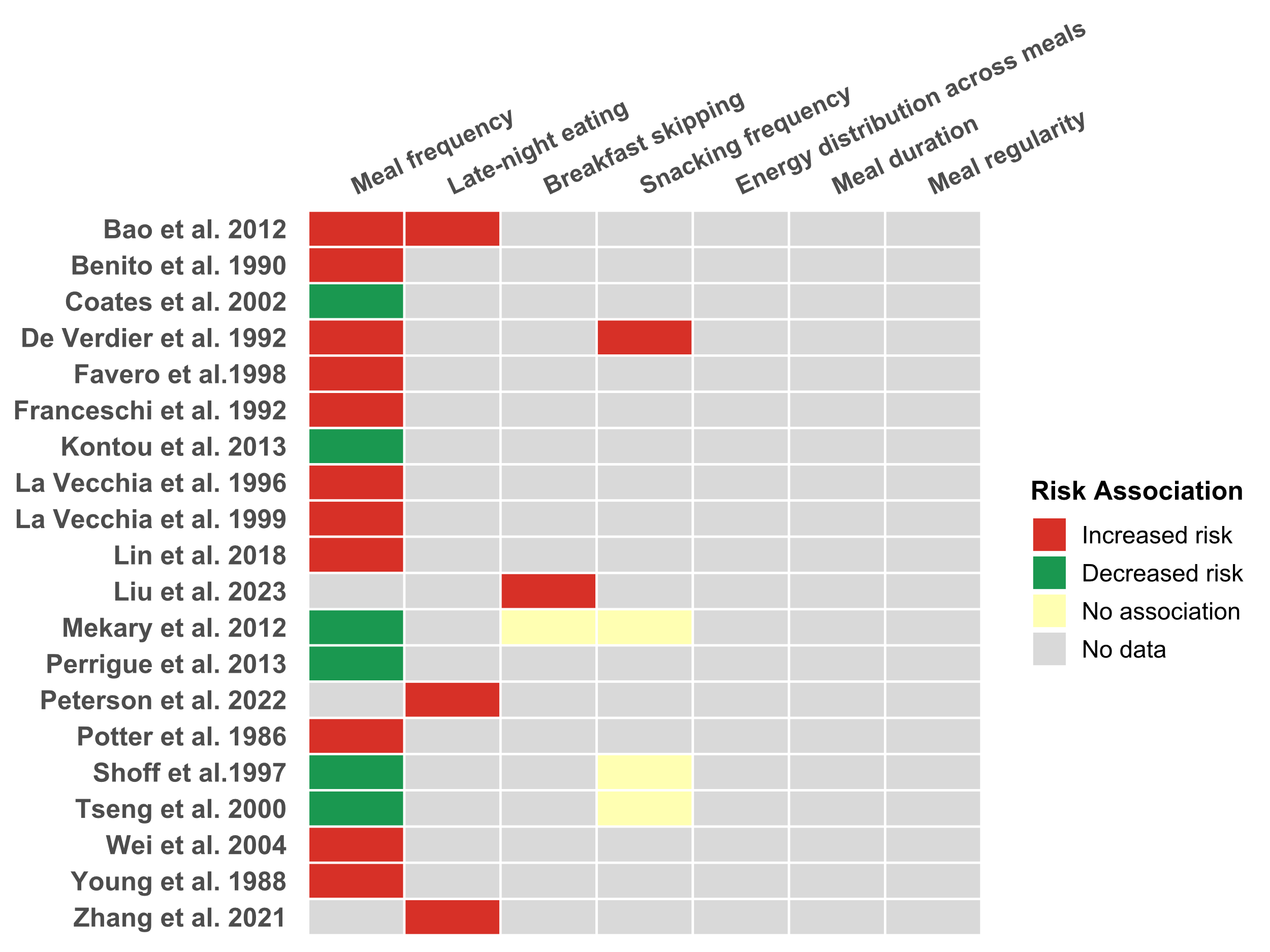

No data

No association

Decrease risk

Increased risk

Keys

Figure 1: Studies that examined temporal eating patterns with CRC risk or mortality that were statistically significant increased risk (red), statistically significant decreased risk( green), no significant associations (yellow), and no data about temporal eating patterns (gray).
